# Navigating Religious and Moral Exemptions in Serving LGBTQ Residents in Long-Term Care Facilities

**DOI:** 10.1093/geroni/igaa057.2343

**Published:** 2020-12-16

**Authors:** Angela Perone

**Affiliations:** University of Michigan, Ypsilanti, Michigan, United States

## Abstract

Almost immediately after the U.S. Supreme Court mandated marriage equality, a surge in policies carved out exceptions to LGBTQ rights for religious or moral reasons. This study examines how long-term care workers, managers, and administrators respond when staff, visitors, or residents challenge LGBTQ rights based on these exemptions. Building on street-level bureaucracy theory, this study employs an advanced multi-method qualitative design with semi-structured staff interviews (n=85), content analysis of facility policies (n=110), and observation of two facilities. Data analysis revealed three key themes: visibility, bodily autonomy/respect, and safety. While nearly all workers expressed universal concern for LGBTQ rights, direct care workers and managers wavered when religious or moral exemptions arose in serving transgender residents. Workers invoked facility policies regarding gender and bodily care to justify differential treatment. This research provides new knowledge and guidance for workers, facilities, and policymakers and theoretical contributions to street-level bureaucracy. Part of a symposium sponsored by Rainbow Research Group Interest Group.

